# Editorial on “A systematic review of the impact of pulmonary thromboendarterectomy on health related quality of life”

**DOI:** 10.1002/pul2.12420

**Published:** 2024-07-29

**Authors:** Alison Witkin

**Affiliations:** ^1^ Pulmonary and Critical Care Medicine Massachusetts General Hospital Boston Massachusetts USA

Pulmonary thromboendarterectomy (PTE) is the treatment of choice for patients with operable chronic thromboembolic pulmonary hypertension (CTEPH) based on the ability to yield dramatic improvements in hemodynamics and association with improved survival.^1^ Multiple guidelines clearly position this as the treatment of choice for CTEPH patients with surgically accessible disease for these reasons. However, in clinical practice, it is not always as straightforward. For example, for a patient with minimal symptoms, the idea of undergoing a major surgery with significant recovery time can be daunting, particularly given the availability of balloon pulmonary angioplasty (BPA) and medical therapy. The World Symposium on Pulmonary Hypertension highlighted the need for research to not only focus on standard clinical trial endpoints but to also evaluate the patient experience.^2^ A better understanding of how these interventions impact not only objective but also subjective patient centered outcomes is necessary to counsel such patients.

In this issue of *Pulmonary Circulation*, Raguragavan et al. report their systematic review on the impact that PTE has on health‐related quality of life (HRQoL). The authors identified 13 studies that examined the effect that PTE has on patients HRQoL in patients with both CTEPH and chronic thromboembolic pulmonary disease (CTEPD). Due to significant heterogeneity in study design, patient population, HR‐QoL score used and likely overlap in patient enrollment between studies, the analysis is limited to a descriptive one. The authors also point out that several of the studies used HRQoL instruments that have not been validated in CTEPH/CTEPD, which limits the strength of the conclusions.

The included studies primarily enrolled patients from Europe and Japan and the majority only included patients with CTEPH who were treated with PTE. This limits the ability to compare HRQoL outcomes based on disease severity, the presence of CTEPD or to other treatment options, such as BPA. All of the included studies that assessed HRQoL pre and post‐PTE showed improvements in HRQoL following PTE, although the magnitude and specifics of these improvements varied between the studies and depending on which tool was used to measure HRQoL. Residual pulmonary hypertension was associated with less improvement in HRQoL while the contribution of preoperative hemodynamics was less clear.

This article provides a detailed summary of the current state of evidence of HRQoL following PTE and demonstrates a clear improvement in HRQoL. However, most of the studies included focus on patients with overt CTEPH who were treated with PTE, where there are multiple guidelines recommending surgical management. The importance of understanding the effects of PTE on HRQoL is perhaps most critical in patients with mild disease or without resting pulmonary hypertension who are at lower risk of significant morbidity and mortality from their chronic pulmonary embolism. While these patients can be successfully treated with PTE or BPA, there is not a consensus on the optimal approach.^3^, ^4^ Additionally, while operability is typically referred to as a binary operable/non‐operable decision, in real world practice, there are borderline patients who may be reasonable candidates for either PTE or BPA. Including HRQoL as an endpoint in future trials and registries, particularly those including patients with CTEPD, would be useful to better understand the potential benefits between different treatment options.

## AUTHOR CONTRIBUTIONS

All aspects of writing and review—Alison Witkin.

## CONFLICT OF INTEREST STATEMENT

Consulting fees from Janssen Pharmaceuticals not directly related to work.

## ETHICS STATEMENT

IRB approval not required.
